# Humoral and cellular immune responses induced by the urease-derived peptide Jaburetox in the model organism *Rhodnius prolixus*

**DOI:** 10.1186/s13071-016-1710-3

**Published:** 2016-07-25

**Authors:** Leonardo L. Fruttero, Natalia R. Moyetta, Augusto F. Uberti, Matheus V. Coste Grahl, Fernanda C. Lopes, Valquiria Broll, Denise Feder, Celia R. Carlini

**Affiliations:** 1 Brain Institute (INSCER) and Graduate Program in Medicine and Health Sciences, Pontifícia Universidade Católica do Rio Grande do Sul, Porto Alegre, RS Brazil; 2 Graduate Program in Cellular and Molecular Biology, Center of Biotechnology, Universidade Federal do Rio Grande do Sul, Porto Alegre, RS Brazil; 3 Department of General Biology l, Insect Biology Laboratory, Universidade Federal Fluminense, Niteroi, RJ Brazil; 4 Department of Biophysics, Biosciences Institute (IB), Universidade Federal do Rio Grande do Sul, Porto Alegre, RS Brazil; 5 Instituto do Cérebro (InsCer) – Pontifícia Universidade Católica do Rio Grande do Sul, Av. Ipiranga 6690, prédio 63, CEP 90610-000 Porto Alegre, RS Brazil

**Keywords:** Cellular immunity, Humoral immunity, Ureases, Jaburetox, *Rhodnius prolixus*

## Abstract

**Background:**

Although the entomotoxicity of plant ureases has been reported almost 20 years ago, their insecticidal mechanism of action is still not well understood. Jaburetox is a recombinant peptide derived from one of the isoforms of *Canavalia ensiformis* (Jack Bean) urease that presents biotechnological interest since it is toxic to insects of different orders. Previous studies of our group using the Chagas disease vector and model insect *Rhodnius prolixus* showed that the treatment with Jack Bean Urease (JBU) led to hemocyte aggregation and hemolymph darkening, among other effects. In this work, we employed cell biology and biochemical approaches to investigate whether Jaburetox would induce not only cellular but also humoral immune responses in this species.

**Results:**

The findings indicated that nanomolar doses of Jaburetox triggered cation-dependent, in vitro aggregation of hemocytes of fifth-instar nymphs and adults. The use of specific eicosanoid synthesis inhibitors revealed that the cellular immune response required cyclooxygenase products since indomethacin prevented the Jaburetox-dependent aggregation whereas baicalein and esculetin (inhibitors of the lipoxygenases pathway) did not. Cultured hemocytes incubated with Jaburetox for 24 h showed cytoskeleton disorganization, chromatin condensation and were positive for activated caspase 3, an apoptosis marker, although their phagocytic activity remained unchanged. Finally, in vivo treatments by injection of Jaburetox induced both a cellular response, as observed by hemocyte aggregation, and a humoral response, as seen by the increase of spontaneous phenoloxidase activity, a key enzyme involved in melanization and defense. On the other hand, the humoral response elicited by Jaburetox injections did not lead to an increment of antibacterial or lysozyme activities. Jaburetox injections also impaired the clearance of the pathogenic bacteria *Staphylococcus aureus* from the hemolymph leading to increased mortality, indicating a possible immunosuppression induced by treatment with the peptide.

**Conclusions:**

In our experimental conditions and as part of its toxic action, Jaburetox activates some responses of the immune system of *R. prolixus* both in vivo and in vitro, although this induction does not protect the insects against posterior bacterial infections. Taken together, these findings contribute to the general knowledge of insect immunity and shed light on Jaburetox’s mechanism of action.

**Electronic supplementary material:**

The online version of this article (doi:10.1186/s13071-016-1710-3) contains supplementary material, which is available to authorized users.

## Background

Ureases (urea amidohydrolases, EC 3.5.1.5) are metalloenzymes that catalyze the breakdown of urea into carbon dioxide and ammonia [1]. They are produced by a wide variety of organisms including bacteria, fungi and plants, but not by animals [2]. Their well-documented enzymatic role results in increased nitrogen availability in a readily usable form and in the alkalization of the medium due to ammonia production. Besides, ureases present biological activities not related to the enzyme function, such as toxicity against fungi and insects as well as exocytosis induction in many cell models [3].

The development of insect resistance and the need for more rational, environment-friendly insecticides are the main driving forces of the research for new substances with entomotoxic properties [4]. In this context, the seed of *Canavalia ensiformis* (Jack Bean) presents at least three urease isoforms that contribute to the plant resistance to the attacks by insects and fungi [3]. When administered orally, ureases are toxic for insects presenting cathepsin-like peptidases in their digestive system (e.g. hemipterans) while insects with digestion based on trypsin-like peptidases (e.g. dipterans) show no susceptibility [5]. This toxicity is explained in part by the fact that digestive cathepsin-like peptidases cleave ureases at specific sites, releasing peptides with insecticidal activity [6–8]. An insecticidal peptide called Pepcanatox was isolated from the in vitro digestion of canatoxin, an isoform of *C. ensiformis* urease [9] and, later, an equivalent recombinant peptide called Jaburetox was produced based on that finding. Jaburetox was shown to be toxic to insects of several orders, irrespective of their digestive enzymes [10–12]. Preliminary reports with transgenic crops such as maize, soybean and sugarcane expressing Jaburetox indicated a higher resistance to the attack of insects (unpublished data). Furthermore, high doses of the peptide are not toxic to mice and rats when given orally [10] making Jaburetox a promising tool for rational insect control. Notwithstanding, the peptide’s toxic mechanism of action in insects is still poorly understood.

Although much simpler than its mammalian counterpart, the host-defense system of insects relies on an intricate array of innate reactions such as complex recognition, signaling and effector systems [13, 14]. Insect immune response can be broadly divided into cellular, including nodulation, encapsulation and phagocytosis, and humoral, which involves nitric oxide (NO), antimicrobial peptides, lysozyme and the phenoloxidase (PO) cascade, among others. Both types of defenses can be recruited simultaneously or separately, depending on the type of insult [15, 16].

It is well established that insect immunity is modulated by eicosanoids [17]. This family of molecules is synthesized from polyunsaturated fatty acids, mainly arachidonic acid, which is released from membrane phospholipids via activation of a phospholipase A_2_ (PLA_2_) [18]. Once free, arachidonic acid then follows diverse enzymatic oxygenation pathways involving cyclooxygenases (COX) to yield prostaglandins and thromboxanes, and lipoxygenases (LOX) producing lipoxins and leukotrienes [19].

*Rhodnius prolixus* is a major insect vector of Chagas disease, an illness that kills approximately 10,000 people annually and affects seven million people worldwide, causing high economic and social costs [20]. Since the foundational studies of Wigglesworth [21], this insect has been a popular model organism for physiological, behavioral and biochemical studies. Furthermore, its genome has been recently sequenced [22], consolidating the species as a powerful tool for genetic and evolutionary approaches.

Previous studies of our group on *R. prolixus* have established that the Jack Bean Urease (JBU) and Jaburetox disturb serotonin-stimulated processes such as diuresis in Malpighian tubules and contraction of the anterior midgut by interfering with eicosanoid signaling [23, 24]. More recently, Defferrari et al. [25] established a link between the toxic effect of JBU and the triggering of immune defenses. Within this context, the aim of this work was to explore the immune responses induced by the toxic urease-derived peptide Jaburetox, using *R. prolixus* as a model. The findings are discussed regarding the current knowledge on the mechanism of action of Jaburetox in insects.

## Methods

### Chemicals

Goat anti-mouse IgG conjugated to Alexa 594 and anti-rabbit IgG conjugated to Alexa 488 antibodies (Molecular Probes Inc., Eugene, OR, USA); mouse anti-α-tubulin monoclonal antibody and 4′,6-diamidino-2-phenylindole (DAPI) (Cell Signalling Technology, Danvers, MA, USA); rabbit anti-Caspase 3 polyclonal antibody, active (cleaved) form and Fluorsave (Merck Millipore, Darmstadt, Germany) were purchased from the indicated commercial sources. The protease inhibitor cocktail and all the remaining reagents were from Sigma-Aldrich (St. Louis, MO, USA).

### Jaburetox production

The recombinant peptide Jaburetox (without a fused V5-antigen) was expressed and purified as described by Lopes et al. [26] with modifications. *Escherichia coli* BL21 (DE3) RIL harboring the plasmid pET23a-Jaburetox was cultured in 20 ml LB (Luria Bertani) Broth, with 100 μg/ml ampicillin and 40 μg/ml chloramphenicol. The culture was grown overnight at 37 °C and 200 rpm. All the content was inoculated in 1 liter of auto induction medium (10 g/l tryptone, 5 g/l yeast extract, 5 g/l glycerol, 3.3 g/l (NH_4_)_2_SO_4_, 6.8 g/l KH_2_PO_4_, 7.1 g/l Na_2_HPO_4_, 0.5 g/l glucose and 2 g/l lactose, with 100 μg/ml ampicillin and 40 μg/ml chloramphenicol) and cultured at 37 °C, 200 rpm, until culture absorbance (A_600_) reached 0.7. The induction conditions were overnight, at 20 °C, 200 rpm. Then, the culture was centrifuged at 8000× *g* for 10 min. The cells were resuspended in 100 ml buffer (50 mM Tris-HCl, pH 7.5, 500 mM NaCl and 5 mM imidazole) and cell suspension was sonicated (20 cycles of 1 min, 20 kHz frequency). The final cell lysate was centrifuged at 14,000× *g* for 40 min and the supernatant was submitted to affinity and size exclusion chromatography according to Lopes et al. [26]. After the purification and before its use for incubations or injections, the peptide (Mr ~11 kDa) was diluted in 20 mM sodium phosphate buffer (PB, pH 7.4).

### Insects

The experiments were conducted with adults and fifth-stage nymphs of *R. prolixus*. The insects were kindly provided by Dr. Pedro Lagerblad de Oliveira and Dr. Hatisaburo Masuda (Institute of Medical Biochemistry, Universidade Federal do Rio de Janeiro, RJ, Brazil) or by Dr. Patricia Azambuja (Instituto Oswaldo Cruz, RJ, Brazil). They were kept under controlled conditions of light:dark cycle (L:D = 12:12 h, lights on at 7.00 a.m.), temperature (27 ± 1 °C) and relative humidity (60 %) in the facilities of UFRGS and fed at regular intervals of three weeks on human blood-filled parafilm-covered acrylic plates maintained at 37 °C on an anodized aluminum-warming table.

### In vitro hemocyte aggregation assays

Hemolymph from fifth-instar nymphs (7 days after blood meal) or adults (3 days after blood meal) was collected with a micropipette from a cut in one of the legs, after previous surface sterilization of the insects with ethanol. The pooled hemolymph was subsequently mixed with cold, sterile, *R. prolixus* saline (150 mM NaCl; 8.6 mM KCl; 2 mM CaCl_2;_ 8.5 mM MgCl_2_; 4 mM NaHCO_3_; 34 mM glucose; 5 mM HEPES, pH 7) [27] at a ratio of 1:1 (*v/v*) in autoclaved tubes. Jaburetox was added to obtain 50, 200 and 500 nM final concentrations in the tubes containing hemolymph and then incubated for 1 h at room temperature (RT) with gentle mixing on an oscillating platform. To evaluate the requirement of extracellular cations, 100 μM Ethylenediaminetetraacetic Acid, Disodium Salt (Na_2_EDTA) was added to the hemolymph/Jaburetox 200 nM mixture and incubated as described. To test the involvement of eicosanoids in the modulation of the aggregation reaction, the following inhibitors of eicosanoid synthesis were employed: dexamethasone; indomethacin (100 μM final concentration each); esculetin and baicalein (300 μM final concentration each). Since the inhibitors’ stock solutions were prepared in 95 % ethanol, controls were performed with equivalent volumes of this solvent. For the remaining experiments, controls without Jaburetox were performed with the addition of the same volumes of PB. The number of cells and aggregates (defined as a cluster of five or more cells following the criteria of [28]) was then determined for each sample by counting in a hemocytometer with a bright field optical microscope [25, 29]. For this and the remaining experiments, the viability of hemocytes was assessed by the Trypan Blue dye exclusion method [30].

### In vivo hemocyte aggregation assays

Unfed fifth-instar nymphs (7 days after ecdysis) weighing an average of 35 mg were employed for all in vivo experiments. Insects were placed ventral side up under a dissecting microscope and injections of Jaburetox (dose of 2 μg/insect) in PB were performed using a microsyringe. Controls were carried out by injecting equivalent volumes of buffer. Six hours after injections, the insects had their surface sterilized by immersion in 70 % ethanol and hemolymph was collected as described. Hemolymph samples were immediately diluted in cold anticoagulant solution (10 mM Na_2_EDTA, 100 mM glucose, 62 mM NaCl, 30 mM sodium citrate, 26 mM citric acid, pH 4.6) at a ratio of 1:5 (anticoagulant: hemolymph) [31]. The number of cells and aggregates was determined as stated above.

### Cell culture and immunocytochemistry

Hemocyte culture was established as in Defferrari et al. [25] with minor modifications. Hemolymph was collected from ethanol-sterilized fifth stage nymphs (7 days after a blood meal) under aseptic conditions and mixed with supplemented Schneider’s insect medium (2 mg/ml tryptose phosphate, 10 % inactivated fetal bovine serum, 0.5 mg/ml glucose, 30 mg/ml L-glutamine, 1× insect medium supplement, 0.04 mg/ml tetracycline, 0.05 mg/ml amphotericin B and 0.05 mg/ml gentamicin, pH 7.0) at a ratio 1:1 (*v/v*). One hundred microliters of the cell suspension (~72,000 hemocytes) were seeded in autoclaved glass cover slips placed in 24-well flat bottom plates and incubated at 24 °C for 1 h. Afterwards, 1 ml of the supplemented culture medium was added and the plates were kept inside an incubator at 24 °C. Cells were viable for at least 10 days with the addition of fresh culture medium every 48 h.

Incubations with 50 and 100 nM Jaburetox were conducted for 24 h and control cells were incubated with equivalent volumes of PB. Hemocytes were washed with *Rhodnius* saline and fixed with 4 % paraformaldehyde for 20 min at RT. For immunofluorescence assays, all incubations were done in PB at RT. After the fixation step, cells were washed three times with PB (5 min each) and permeabilized/blocked with 0.1 % Triton X-100, 5 % Fetal Bovine Serum (FBS) in PB for 30 min. Subsequently, cells were incubated with 1 % FBS plus anti-α-tubulin (1:2000 dilution) as primary antibody, in PB for 1 h. The culture was washed again and a solution with 1 % FBS plus the secondary antibody conjugated to Alexa 594 (dilution 1:400) was added. Controls were performed without the addition of primary or secondary antibodies. Another separated set of cells was stained with 0.1 μg/ml DAPI for 5 min. For both treatments, one final wash was done with distilled water, the slips were air-dried and mounted with Fluorsave. To evaluate apoptosis, another set of cells was blocked and permeabilized as above and incubated with a rabbit anti-activated caspase 3 antibody (dilution 1:50) for 24 h. An anti-rabbit IgG conjugated to Alexa 488 (dilution 1:400) was employed as a secondary antibody and DAPI was used to stain the nuclei. The slides were analyzed under a Zeiss Axiovert 200 inverted fluorescence microscope equipped with an Axiocam MRc camera (Carl Zeiss, Jena, Germany) and the images were acquired using AxioVision Rel 4.8 Software.

### Phagocytosis assay

The phagocytic activity was assessed by incubating hemocytes in culture (previously exposed to PB or Jaburetox for 24 h) with *Saccharomyces cerevisiae* cells labeled with Fluorescein Isothiocyanate (FITC) as described in Figueiredo et al. [32, 33]. Afterwards, hemocytes were rinsed with *Rhodnius* saline, quenched with 1.4 mg/ml trypan blue, fixed and the number of yeasts associated and internalized to the hemolymph cells were counted. The experiment was repeated 3 times, examining 50 hemocytes in each one.

### Phenoloxidase activity assays

Hemolymph samples were collected and diluted ten-fold in hypotonic cacodylate buffer (0.01 M sodium cacodylate, 0.01 M CaCl_2_, pH 7.4). Ten microliters of diluted samples were mixed with 25 μl of the same buffer or 17 μg/ml trypsin (total PO activity) and incubated at 37 °C for 20 min. The reactions were initiated by the addition of 15 μl of a L-DOPA saturated solution (4 mg/ml). The formation of dopachrome was measured at 490 nm in a SpectraMax M3 plate reader (Molecular Devices Inc., Downingtown, PA, USA). The readings were monitored at 37 °C for 1 h and the PO activity was expressed as A_490_ × 100 [34] for spontaneous and total (in vitro, trypsin-activated) activities.

### Nitric Oxide Synthase (NOS) activity assays

For these experiments, the insects were injected as described above and 6 h afterwards the hemolymph was collected individually and homogeneized in 20 mM Tris-HCl (pH 7.4), 0.32 M sucrose, 2 mM Na_2_EDTA, 2 mM dithiothreitol (DTT) and 10 % protease inhibitors. The homogenates were centrifuged (10,000× *g*, 10 min, 4 °C) and the protein concentration was determined by Bradford [35]. NOS activity was measured as described in Galvani et al. [36], incubating the samples at 37 °C in a reaction mixture containing 50 mM PB (pH 7.0), 1 mM CaCl_2_, 1 mM L-arginine, 100 μM NADPH, 10 μM DTT, 0.1 μM catalase, 4 μM superoxide dismutase (SOD) and 5 μM oxyhemoglobin. Formation of methemoglobin was monitored at 401 nm. Controls were done carrying out the reaction in the presence of the NOS inhibitor NG-methyl-L-arginine (L-NMMA, 1 mM).

### Lysozyme activity assay

The fluorogenic substrate 4-methylumbelliferyl-*β*-D-*N*, *N’*, *N”*-Tetraacetylchitotriose was employed to evaluate lysozyme activity in hemolymph samples of injected insects following the manufacturer’s instructions. For the assay 5 μl of a 0.8 mM solution of the substrate were added to 94 μl of 10 mM PB (pH 5.5) and incubated for 5 min at 37 °C. Subsequently, 1 μl of the diluted hemolymph was added and the mixture was incubated for 20 min at 37 °C. The reaction was stopped by the addition of 100 μl of 500 mM Na_2_CO_3_ and the readings were performed in the plate reader set for 355 and 460 nm as the excitation and emission wavelengths, respectively. The specific enzyme activity was expressed as Relative Fluorescence Units (RFU)/μg protein/20 min.

### Antibacterial assays

The induction of humoral antibacterial response upon Jaburetox injection was assessed by inhibition zone assays in agar plates, as described by Feder et al. [37] with modifications. *Escherichia coli* ATCC 25922 cells were grown aerobically in LB medium at 37 °C and used to seed agar plates. Six hours after PB or Jaburetox injections, the hemolymph was collected and mixed (1:1 *v/v*) with cold, sterile anticoagulant solution and centrifuged to pellet the hemocytes (10,000× *g,* 10 min, 4 °C). The supernatant was recovered and stored at -80 °C until use, after protein determination [35]. For the assays of antibacterial activity, samples (2 μl) of diluted hemolymph were applied to the bacteria-coated agar plates. Inhibition zones around the wells were analyzed after 24 h keeping the plates at RT. Cecropin A and chloramphenicol were used as positive inhibition controls.

Turbidimetric assays were carried out according to Castro et al. [38] and Vieira et al. [39] with minor modifications. Briefly, the collected hemolymph was diluted 1:1 *v/v* in ultrapure water and incubated with an *E. coli* suspension under agitation at 37 °C for 10 h. Controls were performed: (i) with bacteria and without hemolymph; and (ii) with bacteria and antibiotics. The absorbance at 550 nm was monitored every hour. All data points were blanked against time zero to account for the opacity of the samples.

In another set of experiments, 5 h after receiving the first injection of either PB or Jaburetox, insects were subsequently inoculated with ~1.8 × 10^6^ Colony-Forming Units (CFU) of *Staphylococcus aureus* ATCC 25923 (grown aerobically in LB medium at 37 °C). Five hours afterwards, the hemolymph was collected and the number of CFU in each insect’s hemolymph was determined by the drop-plate method.

### Statistical analysis

For the aggregation experiments as well as for phagocytosis and turbidimetric assays, hemolymph pools of 6–8 insects were employed, whereas for the remaining assays the insects were processed individually. One-way parametric ANOVA was used for comparisons between means and the Student-Newman-Keuls was employed as a *post-hoc* test for the in vitro aggregation and phagocytosis experiments. Student’s *t*-test was used for analysis of PO, NOS and lysozyme activities as well as for turbidimetric assays, while the non-parametric Mann-Whitney *U*-test was employed for the in vivo aggregation and bacteria clearance experiments. Graphs and statistical tests were performed using the software products GraphPad Prism 5 and GraphPad Instat 3.0 (San Diego, CA, USA). A *P* value < 0.05 was considered statistically significant and the results were expressed as mean ± standard error of the mean (SEM). All experiments were performed at least in triplicates and the *n* for each assay is indicated in the corresponding figure captions.

## Results and discussion

### Jaburetox induced hemocyte aggregation in vitro

**Fig. 1:**
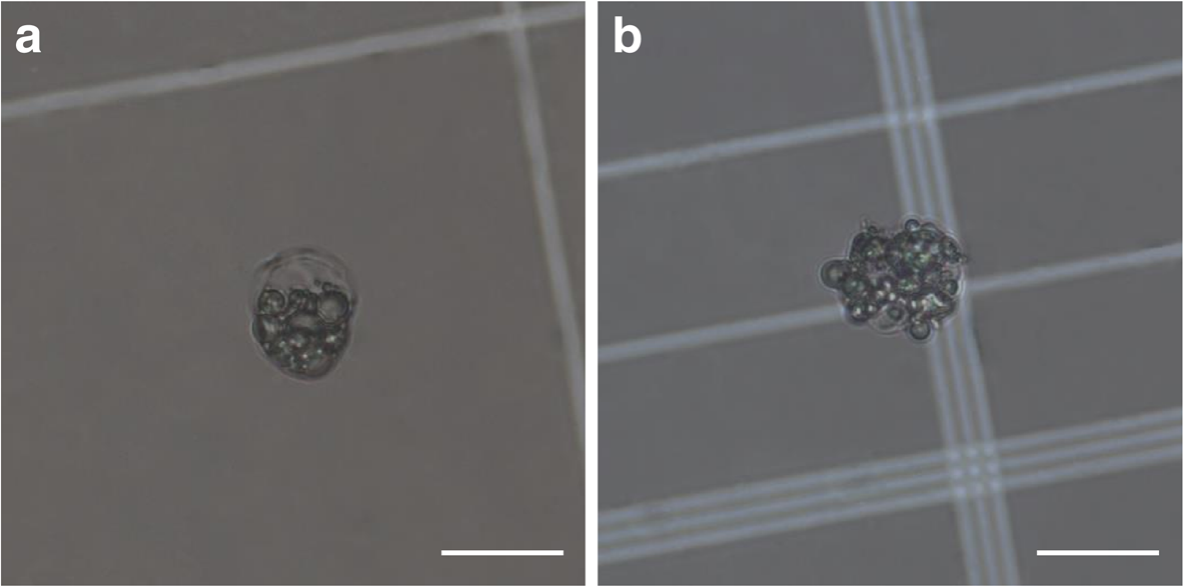
In vitro Jaburetox-induced aggregates. *R. prolixus* hemolymph was collected, diluted with *Rhodnius* saline and incubated with different concentrations of Jaburetox for 1 h at room temperature. **a**, **b** Bright-field images of representative aggregates as seen in the hemocytometer (representative experiments of three independent assays). *Scale-bars*: 50 μm (magnification of 400×)

**Fig. 2:**
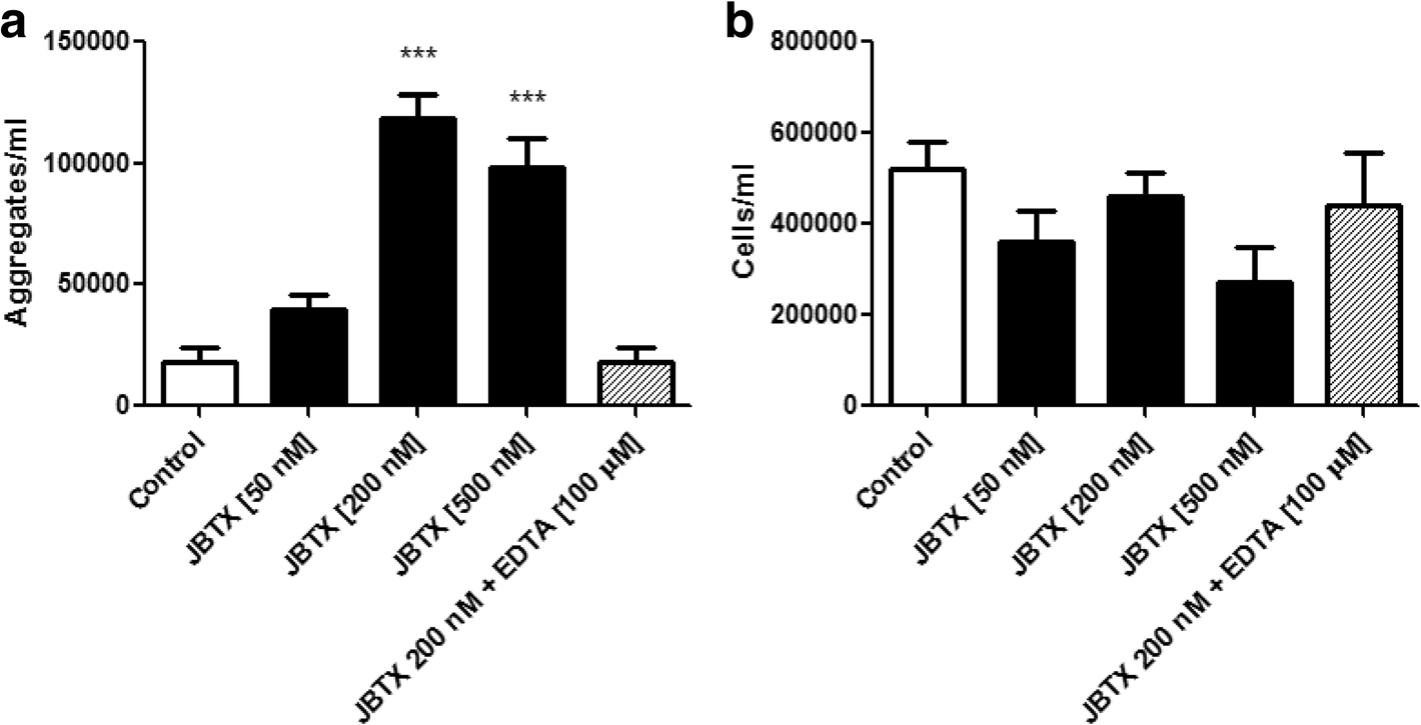
Effect of EDTA and dose-dependence of Jaburetox-induced in vitro aggregation of hemocytes. Hemolymph was collected, diluted with *Rhodnius* saline and incubated with different concentrations of Jaburetox alone or Jaburetox plus 100 μM of Na_2_EDTA for 1 h at room temperature. Controls were performed employing equivalent volumes of saline. The number of aggregates (**a**) and free cells (**b**) was counted using a hemocytometer. The values are expressed as the number of aggregates or the number of free cells per ml of hemolymph. Mean ± SEM (*n* = 3 for controls and *n* = 5 for Jaburetox-treated insects). ***P < 0.0001 *vs* control

In vitro assays of hemocytes aggregation were first undertaken to investigate if Jaburetox would induce an immune response in *R. prolixus*. This versatile approach, previously employed for plant ureases (JBU in [25] and soybean urease, unpublished results), allows not only a rapid assessment of cellular immune activation, but also enables the use of specific inhibitors that ultimately provide information of the modulation pathways involved. In this work, when the hemolymph of fifth-instar nymphs was collected and incubated with different concentrations of Jaburetox for 1 h at RT, the number of aggregates (Fig. 1) increased for all tested concentrations (i.e. 50, 200 and 500 nM, Fig. 2a) (ANOVA: *F =* 46.20, *df* = 22, *P* < 0.0001) rising from ~18,000 aggregates/ml of hemolymph in the control to ~118,000 aggregates/ml of hemolymph for 200 nM Jaburetox. The number of freely suspended cells did not vary significantly among the treatments (ANOVA: *F =* 2.09, *df* = 22, *P* = 0.125) (Fig. 2b). The addition of 100 μM of the chelating agent Na_2_EDTA inhibited the Jaburetox-induced hemocyte aggregation (Fig. 2a), indicating that this immune response is dependent on extracellular cations, probably calcium. The need of divalent cations for the aggregation can be related either to the calcium dependence of some insect PLA_2_ [40], the enzyme that catalyzes the first step in eicosanoids biosynthesis, or specifically to the mechanism of action of ureases and derived peptides. In line with the latter, the requirement of divalent cations (calcium) in several biological activities mediated by ureases has been reported previously, including assays of JBU-induced diuresis inhibition in *R. prolixus* Malpighian tubules [23] and platelet activation by the *C. ensiformis* urease canatoxin [41], and by the microbial ureases of *Bacillus pasteurii* [42] and of *Helicobacter pylori* [43].

**Fig. 3:**
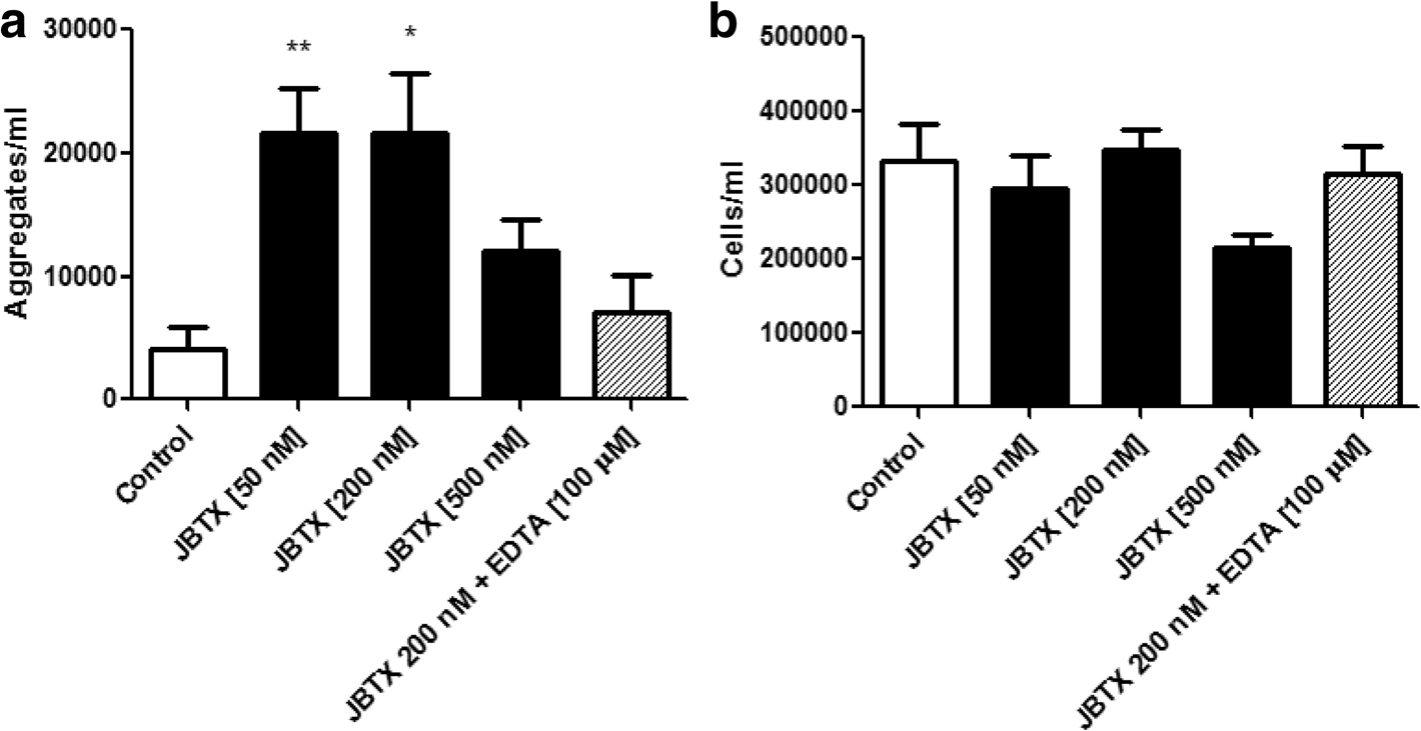
Jaburetox induced in vitro aggregation of hemocytes from *R. prolixus* adults 3 days after a blood meal. Hemolymph was collected, diluted with *Rhodnius* saline and incubated with different concentrations of Jaburetox alone or Jaburetox plus 100 μM of Na_2_EDTA, for 1 h at room temperature. Controls were performed employing equivalent volumes of saline. The number of aggregates (**a**) and free cells (**b**) was counted using a hemocytometer. The values are expressed as the number of aggregates or the number of free cells per ml of hemolymph. Mean ± SEM (*n* = 4 for controls and *n* = 6 for Jaburetox-treated insects). **P < 0.01 *vs* control, *P < 0.05 *vs* control

Taking into account that different stages of insects respond differently to immune challenges and that *R. prolixus* adults are resistant to canatoxin, whereas nymphs are not [5, 6, 9], the aggregation assay was performed in vitro with hemolymph obtained from adults (Fig. 3). In this case a lower dose of Jaburetox (50 nM) triggered a statistically significant aggregation response, increasing from ~4000 aggregates/ml in the hemolymph of control insects to ~21,700 aggregates/ml of hemolymph for 50 and 200 nM Jaburetox treatments (ANOVA: *F =* 6.02, *df* = 27, *P* = 0.018) (Fig. 3a). Interestingly, a higher Jaburetox concentration of 500 nM did not induce a significant difference in the number of aggregates when compared to control (Fig. 3a). This can be explained either because this high dose of Jaburetox led to a greater mortality of the cells (rendering less available cells to aggregate) and/or because more cells are clumped in groups smaller than what we considered an aggregate, i.e. in groups of 2–4 cells. As occurred in the previous assays with hemolymph from nymphs, no statistically significant differences were observed in free cells for the different treatments (ANOVA: *F =* 1.74, *df* = 27, *P* = 0.175) (Fig. 3b). Na_2_EDTA (100 μM) prevented the Jaburetox-induced aggregation (Fig. 3a), indicating the need of extracellular cations for this immune reaction. Thus in agreement to the observation made by Defferrari et al. [25] for JBU-induced responses, the Jaburetox-elicited in vitro activation of the cellular immunity (Figs. 2 and 3) is also cation-dependent. Previous reports employing *Triatoma infestans* [11] and experiments with *R. prolixus* (unpublished results) showed that, upon Jaburetox treatment, adult insects present a reduced percentage of mortality and/or longer times of survival as compared to nymphs. In line with those findings, here we have observed that a statistically significant hemocyte aggregation is achieved with 50 nM Jaburetox in adults and 200 nM Jaburetox in fifth-instar nymphs (Figs. 2 and 3). Feder et al. [37] demonstrated that an impaired capacity to produce nodules was related to immune depression; therefore, it is plausible that the ability of the adults to engage in an early cellular response makes them more resistant to Jaburetox.

**Fig. 4:**
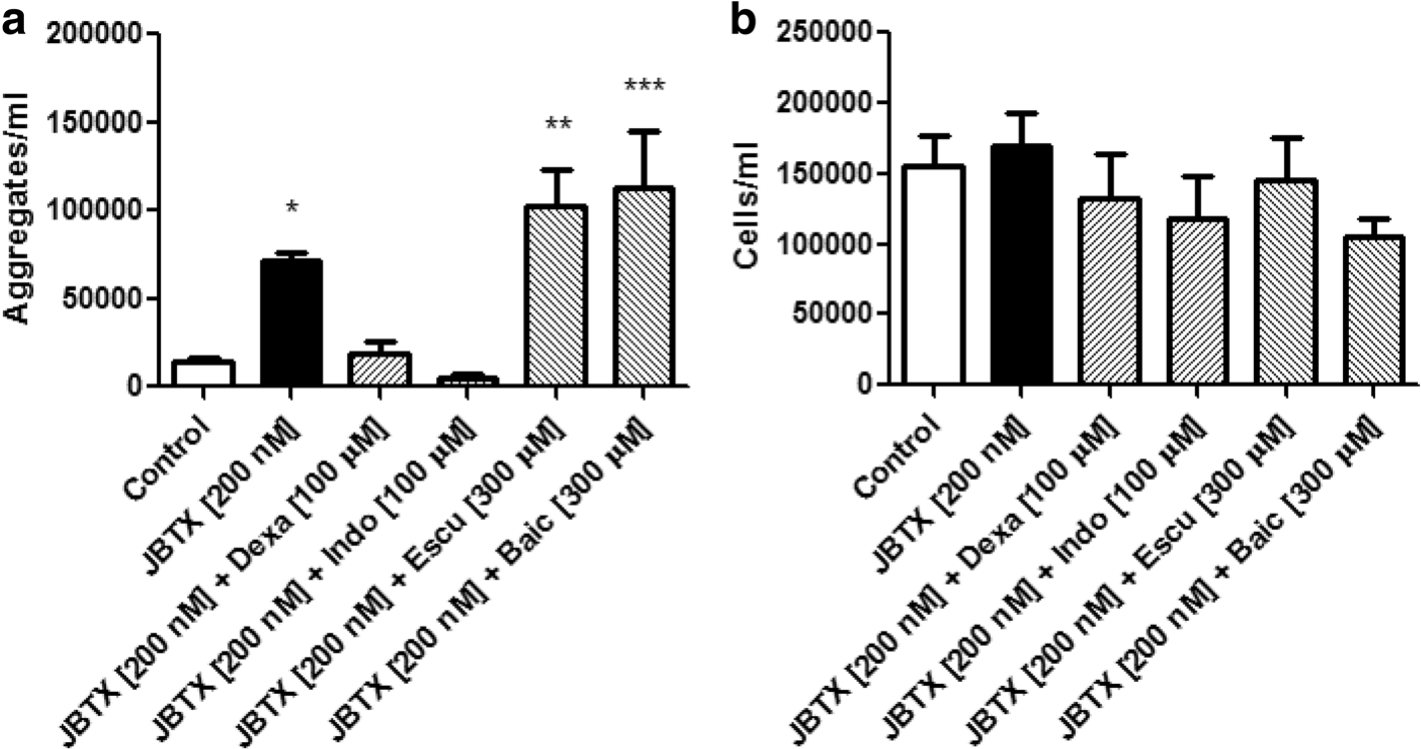
Effect of inhibitors of eicosanoid synthesis on the in vitro Jaburetox-induced hemocyte aggregation. Hemolymph was collected, diluted with *Rhodnius* saline and incubated with different concentrations of Jaburetox alone or Jaburetox plus the indicated concentrations of dexamethasone (dexa), indomethacin (indo), esculetin (escu) or baicalein (baic) for 1 h at room temperature. Controls were performed employing equivalent volumes of saline and ethanol. The number of aggregates (**a**) and free cells (**b**) was counted using a hemocytometer. The values are expressed as the number of aggregates or the number of free cells per ml of hemolymph. Mean ± SEM (*n* = 4 for controls and *n* = 6 for Jaburetox-treated insects). ***P < 0.0001 *vs* control; **P < 0.01 *vs* control; *P < 0.05 *vs* control

For the remaining experiments, we chose to focus on fifth-instar to facilitate comparisons to our previous work [25]. To test the effect of eicosanoid synthesis inhibitors, the in vitro aggregation assay was performed adding dexamethasone (PLA_2_ inhibitor) [44]; indomethacin (a COX-1 and COX-2 inhibitor) [45] as well as baicalein (12-LOX inhibitor) [46] and esculetin (5-LOX and 12-LOX inhibitor) [47]. Dexamethasone and indomethacin at concentrations of 100 μM inhibited Jaburetox-induced aggregation while esculetin and baicalein at concentrations of 300 μM did not (ANOVA: *F =* 9.86, *df* = 33, *P* < 0.0001) (Fig. 4a). Once again, the number of free cells was not significantly changed by the treatments (ANOVA: *F =* 0.96, *df* = 33, *P* = 0.461) (Fig. 4b). These results indicate that Jaburetox-induced aggregation of hemocytes is mediated by the COX pathway, similar to what was reported for the JBU-induced effect [25]. Defferrari and co-workers [48] also demonstrated that the product of a PLA_2_ XII gene is involved in the lethal effect of JBU in *R. prolixus*, since knocking down this gene significantly reduced the JBU-associated toxicity. The involvement of the COX pathway (prostaglandins) in the modulation of insect immunity is well established [17]. On the other hand, there are reports indicating that LOX products are produced by isolated hemocytes [49] and have effects in whole insects [50]. Our results showed that the treatment with LOX inhibitors plus 200 nM Jaburetox increased the number of aggregates when compared to controls incubated with 200 nM Jaburetox alone (Fig. 4a), suggesting a possible involvement of the LOX pathway in *R. prolixus* aggregation reaction. An alternative explanation is that, in the presence of LOX inhibitors, more substrate is available for the COX pathway. In contrast to what was reported in insects, the activation of different types of mammalian cells by ureases from various sources depends on LOX products [42, 43, 51–54].

### Jaburetox induced hemocyte aggregation in vivo

**Fig. 5:**
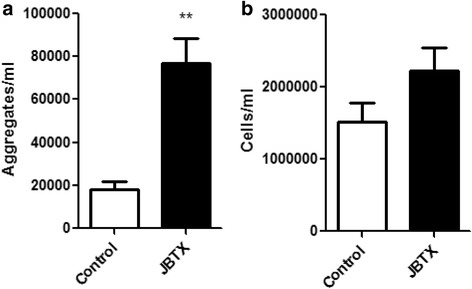
Jaburetox induced in vivo hemocyte aggregation. Fifth-instar nymphs (7 days after ecdysis) were injected with PB (controls) or with Jaburetox in a dose of 2 μg/insect. Six hours later, the hemolymph was collected and diluted with anticoagulant solution. The number of aggregates (**a**) and free cells (**b**) was counted using a hemocytometer. The values are expressed as the number of aggregates or the number of free cells per ml of hemolymph. Mean ± SEM (*n* = 4 for controls and *n* = 6 for Jaburetox-treated insects). **P < 0.01 *vs* control

The insecticidal effect of Jaburetox injected into *R. prolixus* [11, 12] as well as in the related triatomine *T. infestans* [11, 36] was demonstrated elsewhere. Here, in vivo experiments were performed using Jaburetox injections at ~0.06 μg Jaburetox/mg of insect (2 μg Jaburetox/insect) and the hemolymph was collected 6 h afterwards. This dose was chosen due to the fact it takes 96 h to kill the insects [12], allowing sampling of the hemolymph at 6 h. As shown in Fig. 5a, the injection of Jaburetox triggered aggregation of the hemocytes, increasing from a basal level of ~18,000 aggregates/ml of hemolymph in controls to ~77,000 aggregates/ml of hemolymph in the Jaburetox-treated insects (Mann-Whitney U = 0.0000, *P =* 0.008). As in in vitro experiments, the number of free cells was not significantly modified by the treatments (Mann-Whitney U = 7.0000, *P =* 0.177) (Fig. 5b).

### Changes induced by Jaburetox in cultured cells

**Fig. 6:**
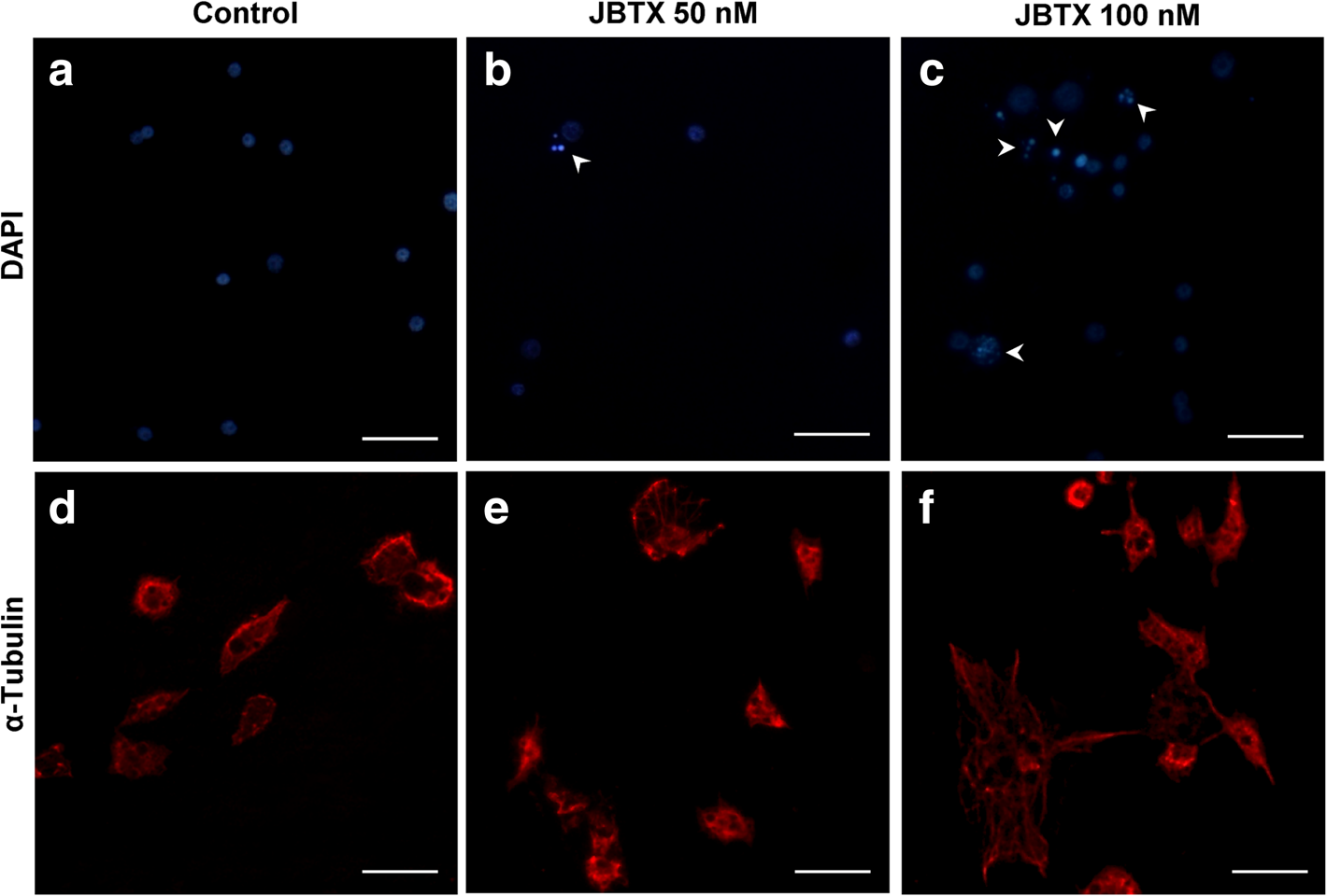
Jaburetox-induced modifications in cultured hemocytes. The hemolymph was collected and the hemocytes were cultured in a supplemented Schneider’s insect medium as described in Methods. Cells were incubated for 24 h with 50 nM Jaburetox (**b**, **e**), 100 nM Jaburetox (**c**, **f**) or an equivalent volume of PB (**a**, **d**) as control and stained with DAPI (**a-c**) or with anti-α-tubulin/Alexa 594 (**d-f**). *Arrowheads* indicate condensed nuclei. The figure shows representative experiments of three independent assays. *Scale-bars*: 30 μm (magnification of 1000×)

**Fig. 7:**
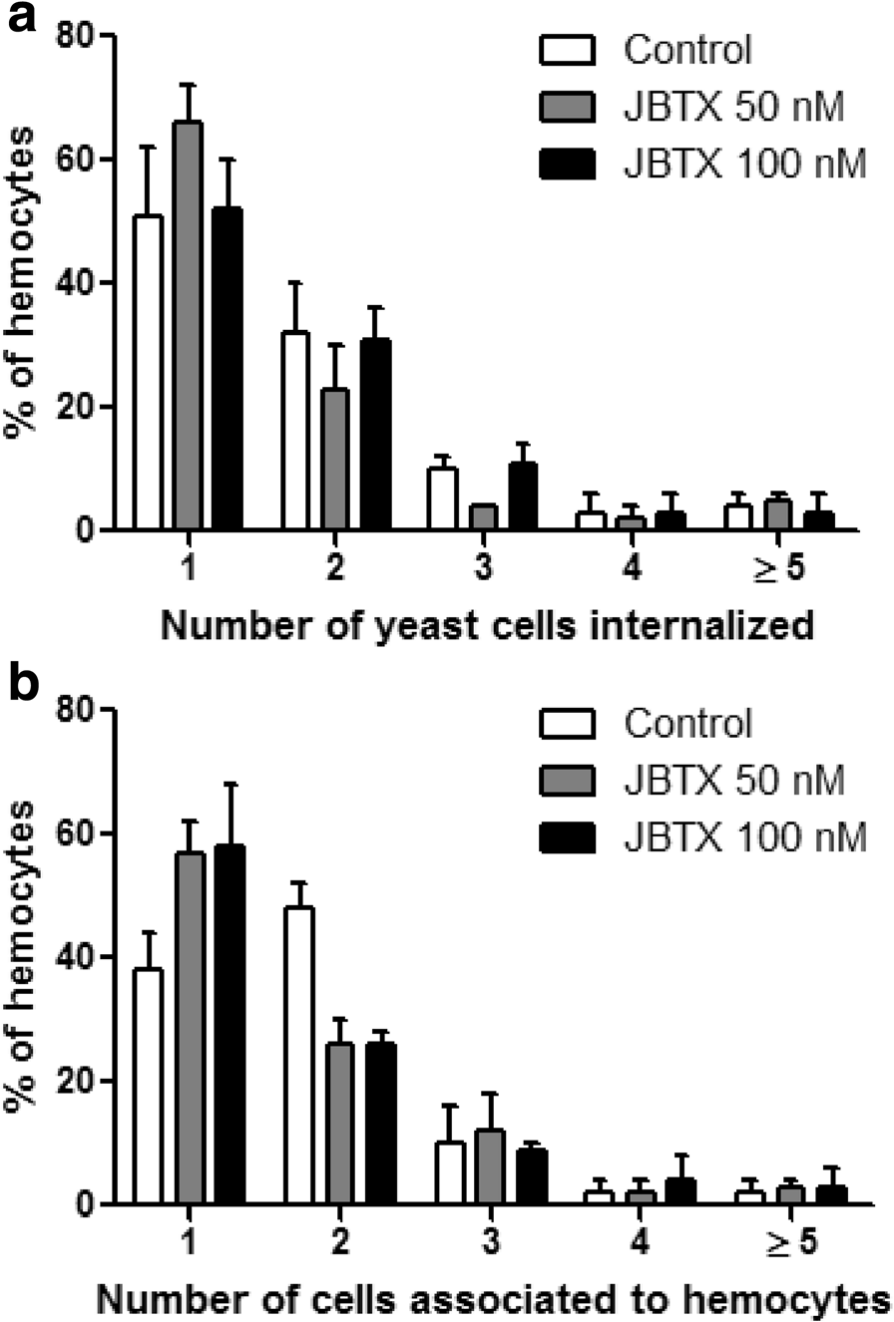
Phagocytosis assay in cultured hemocytes. Hemocytes were cultured as described in Methods and incubated with PB (Control) or with 50 and 100 nM Jaburetox for 24 h. Afterwards, the hemocytes were incubated with 1 × 10^7^ FITC conjugated yeast cells/ml for 1 h and the yeast cells internalized by the hemocytes (**a**) and associated to them (**b**) were counted. The values represent Mean ± SEM and the experiment was repeated 3 times

**Fig. 8:**
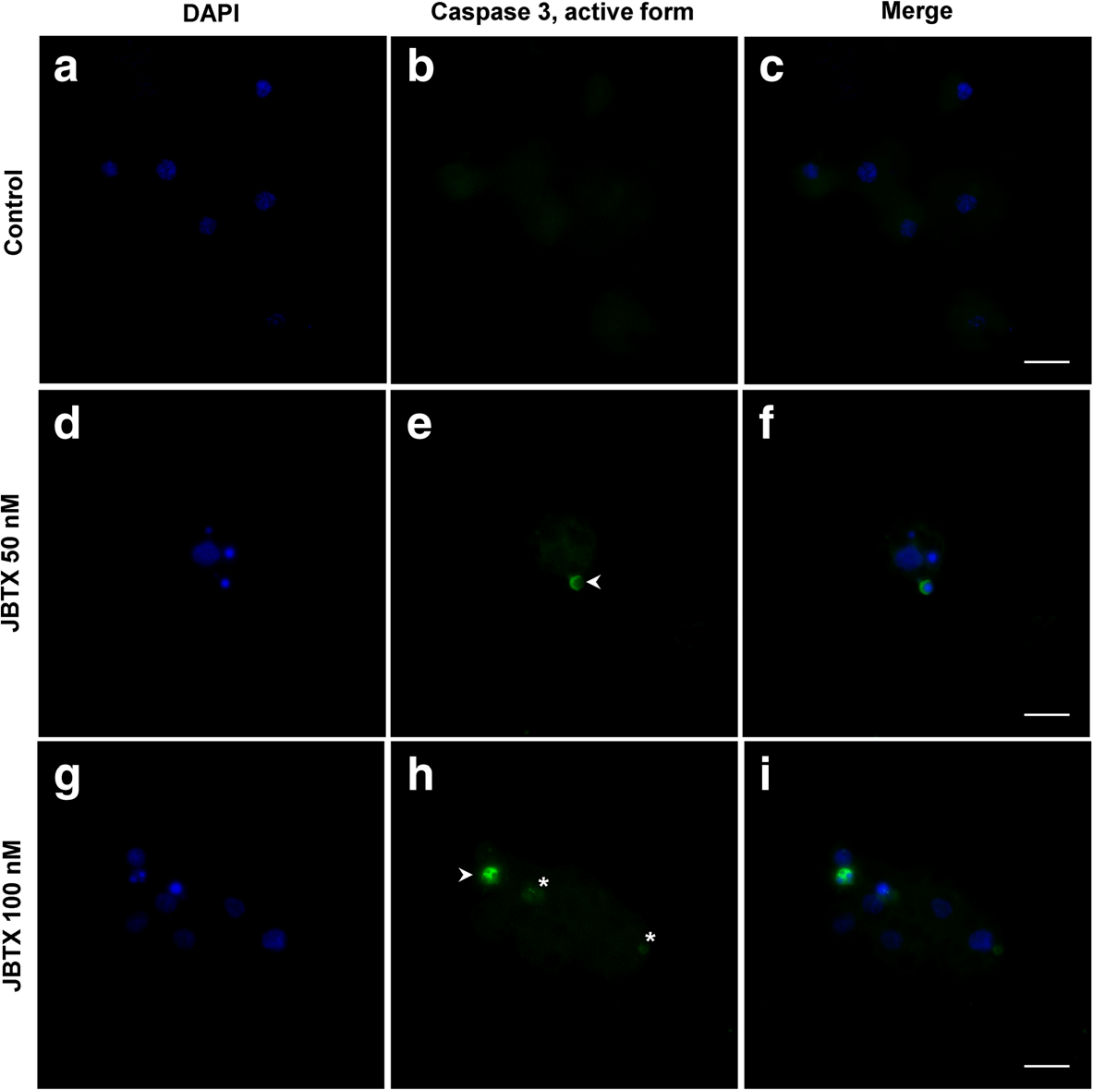
Detection of active caspase 3 in cultured hemocytes. Cultured hemocytes incubated with PB (control) (**a**-**c)** or with 50 (**d**-**f**) and 100 nM (**g**-**i**) Jaburetox for 24 h were stained with the anti-cleaved caspase 3 antibody (**b**, **e**, **h**) and counterstained with DAPI (**a**, **d**, **g**). *Arrowheads* indicate cells positive for cleaved caspase-3 while *asterisks* point to cells with incipient positive signal for the antibody. The figure shows representative experiments of three independent assays. *Scale-bars*: 10 μm (magnification of 1000×)

Cultured hemocytes showed dose-dependent modifications upon 24 h of incubation with Jaburetox. At 50 nM, Jaburetox caused chromatin condensation in a few cells (~8 %, Fig. 6b) when compared to the control (Fig. 6a) and this effect was more pronounced for the 100 nM Jaburetox dose (~36 % of the cells, Fig. 6c). These results were similar to those reported by Defferrari et al. [25] observing chromatin condensation of hemocytes exposed to 100 nM JBU, but not seen at 50 nM. The cytoskeleton was affected by the addition of the toxic peptide to the incubation medium (Fig. 6d-f). Jaburetox (50 nM) caused a slight, but detectable, tubulin disorganization, seen as a larger spreading of the cells (Fig. 6e). This phenomenon was more evident at a 100 nM Jaburetox (Fig. 6f) when compared to controls (Fig. 6d). However, it is noticeable in Fig. 6 that only a subset of hemocytes seem to be affected by the peptide. Thereafter, we performed assays to investigate if the phagocytic activity of the cultured cells was altered by the Jaburetox treatment. Considering the cells that displayed phagocytic activity, no significant differences were observed in the number of fluorescent yeast cells internalized (ANOVA: *F =* 0.14–3.31, *df* = 8, *P* = 0.174–0.875) (Fig. 7a) or associated (ANOVA: *F =* 0.29–5.80, *df* = 8, *P* = 0.066–0.813) (Fig. 7b) to the hemocytes. Programmed cell death of hemocytes is a strategy developed by some insect species to withstand infections, as is the case of autophagy activation in response to bacterial challenge [55] and apoptosis triggering as an effective way to reduce baculoviruses replication and spreading [56]. The involvement of apoptosis in the Jaburetox-mediated mechanism of action was demonstrated by the employment of an anti-active caspase 3 antibody (Fig. 8). Some of the cells positive for active caspase 3 also showed chromatin condensation (Fig. 8d-f and Fig. 8g-i). It is thus probable that Jaburetox triggers programmed cell death in a subset of hemocytes and we are currently exploring this possibility using specific markers. While there is little information on the cytotoxic effects that Jaburetox might cause in mammalian systems, the available data point to different actions of ureases regarding cell death in mammal models. No acute cytotoxic effects of JBU, *B. pasteurii* or *H. pylori* ureases could be seen in blood platelets, pancreatic b-cells or macrophages (reviewed in [3]). However, long-term incubations in the presence of canatoxin led to cytolytic effects in rodent and human cell cultures [57]. In contrast, the urease of *H. pylori* inhibited apoptosis of human neutrophils [54].

Preliminary immunofluorescence assays suggest that the Jaburetox is internalized by cultured hemocytes (unpublished results) and co-localization studies are underway to establish the precise subcellular location of the peptide as well as to determine the time course of its processing.

### Jaburetox triggered a humoral immune response in vivo

**Fig. 9:**
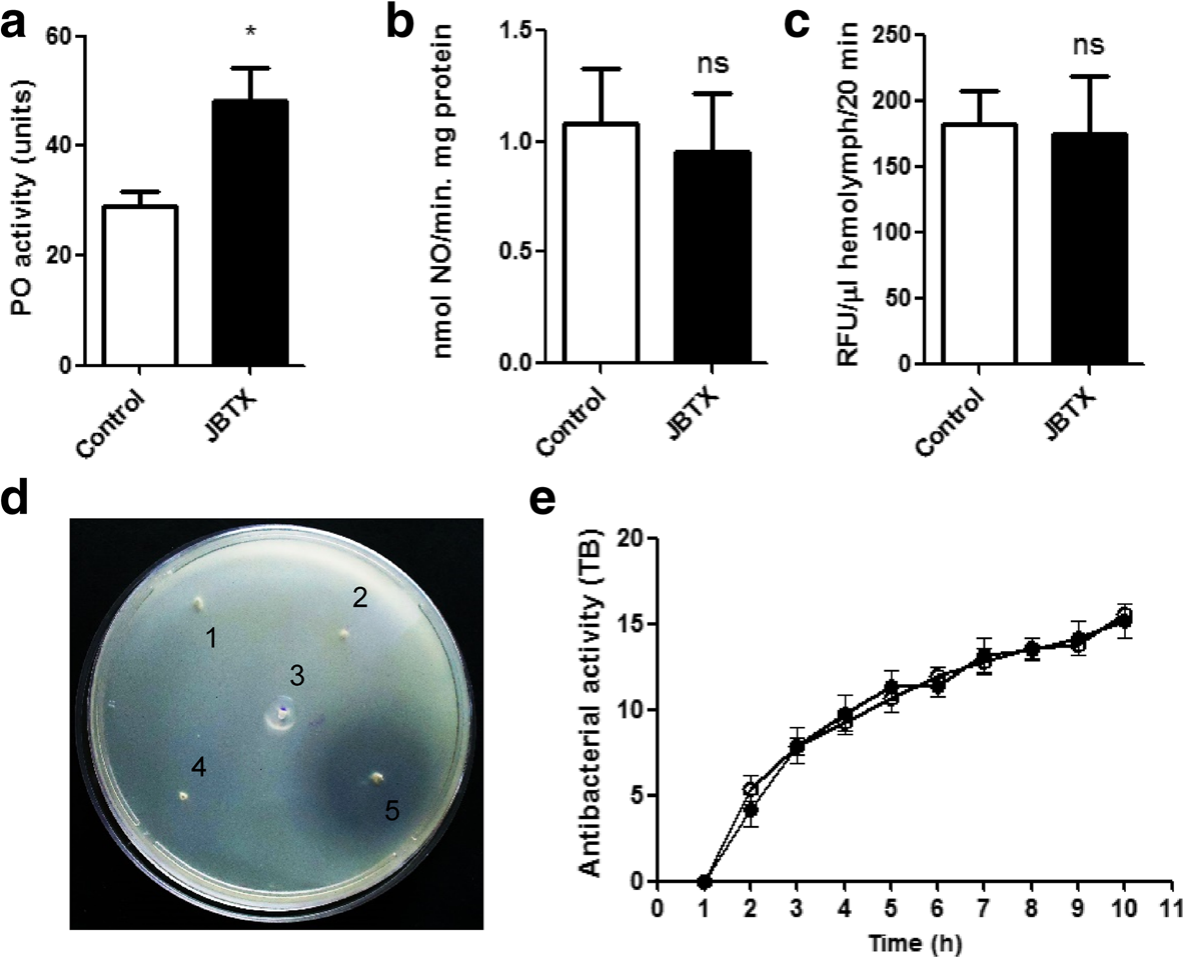
Jaburetox induced an immune humoral response in vivo. Insects were injected with PB (controls) or with Jaburetox in a dose of 2 μg/insect. Six hours later, the hemolymph was collected and assayed. **a** Phenoloxidase activity was measured using L-DOPA as substrate. The values are expressed as phenoloxidase activity (A_490_ × 100) and are the mean ± SEM (*n* = 5). *P < 0.05 *vs* control. **b** NOS activity was determined using oxyhemoglobin as substrate. The values are expressed as nmol NO/min.mg protein and are the mean ± SEM (*n* = 6) (ns, no statistically significant difference). **c** Lysozyme activity was assessed employing the fluorogenic substrate 4-methylumbelliferyl-*β*-D-*N*, *N*’, *N*”-Tetraacetylchitotriose and are the mean ± SEM (*n* = 4) (ns, no statistically significant difference). **d** Antibacterial cecropin-like activity in plates seeded with *E. coli* ATCC 25922. Two μl of each sample were added in holes punched in the agar plate and the inhibition zone were recorded after and incubation of 24 h at room temperature. 1,2: PB-injected samples (negative controls); 3,4: Jaburetox-injected samples; 5: chloramphenicol (40 μg/ml, positive control). **e** Turbidimetric assay incubated for 10 h with readings at each hour. (○) indicate control and (●) Jaburetox-treated insects. The results are expressed as antibacterial activity (TB) and are the mean ± SEM (*n* = 4)

PO are key enzymes that mediate steps of the melanization reaction, which is fundamental for the sclerotization of a new cuticle after ecdysis as well as for the encapsulation of pathogens in the hemolymph [58]. These enzymes also produce quinones that can kill microorganisms directly and participate in immune cellular defenses such as phagocytosis. PO are synthesized as zymogens or prophenoloxidases (proPO), which are activated by serine proteases upon recognition of a pathogen associated molecular pattern (PAMP) and activation of the serine protease cascade [59]. To test if Jaburetox would induce the activation of this enzyme upon the in vivo treatment, we performed Jaburetox injections as described for the in vivo hemocyte aggregation assay and measured hemolymph PO activity using L-DOPA as substrate. As observed in Fig. 9a, the injection of Jaburetox increased PO spontaneous activity in more than 70 % when compared to controls injected with PB alone (*t*-test: *t =* 2.88, *df =* 8, *P* = 0.021). No significant differences were registered for total trypsin-activated PO activity (80.6 ± 7.2 units for the control *vs* 92.5 ± 10.1 units for the Jaburetox-treated insects (*t*-test: *t =* 1.69, *df =* 8, *P* = 0.165). These results indicate that the Jaburetox treatment not only triggers the cellular immune response but also the humoral one. It is well established that the PO cascade is a key humoral response to insults in insects and is mediated by eicosanoids [60]. In our work, the increase in PO activity upon Jaburetox treatment (Fig. 9a) is in line with the outcome of the experiments using eicosanoid inhibitors (Fig. 4) and with reports from other groups showing that COX-derived eicosanoids also signal to proPO release and activation in *Spodoptera exigua* [61], *Galleria mellonella* [62] and *R. prolixus* [63].

NOS is the enzyme responsible for the synthesis of NO, a metabolite implicated in nitrinergic signaling and involved in defense because of its toxicity to pathogens [64, 65]. In *R. prolixus*, Gazos-Lopes et al. [66] demonstrated that the NOS activity of salivary glands is diminished in response to *Trypanosoma rangeli* infection. Our group has previously described [36] that Jaburetox injections decreased NOS activity in the central nervous system of *T. infestans*. Nevertheless, in the present work, injections of the toxic peptide did not induce changes in NOS activity (*t*-test: *t =* 0.36, *df =* 10, *P* = 0.723) (Fig. 9b), indicating that the effect is tissue-specific.

Cecropin-like and lysozyme activities are humoral responses that occur mainly upon bacterial challenge, although lysozyme activity is also related to other types of pathogens (parasites for example, reviewed in [67]). As shown in Fig. 9c, the levels of lysozyme activity as determined using a fluorogenic substrate showed no differences between the PB-injected controls and Jaburetox-treated samples (*t*-test: *t =* 0.24, *df =* 8, *P* = 0.822). Likewise, the challenge with Jaburetox did not induce antibacterial cecropin-like activity since no inhibition zone was detected in any of the treatments with hemolymph collected at six hours post-injection (Fig. 9d and Additional file 1: Figure S1). In line with those findings, turbidimetric assays incubating hemolymph from control and Jaburetox-treated insects with bacteria for 10 h at 37 °C (Fig. 9e), revealed no significant differences between the treatments (*t*-test: *t =* 0.07–1.06, *df =* 6, *P* = 0.332–0.949).

### Jaburetox reduced bacterial clearance and survival of *R. prolixus* upon bacterial infection

**Fig. 10:**
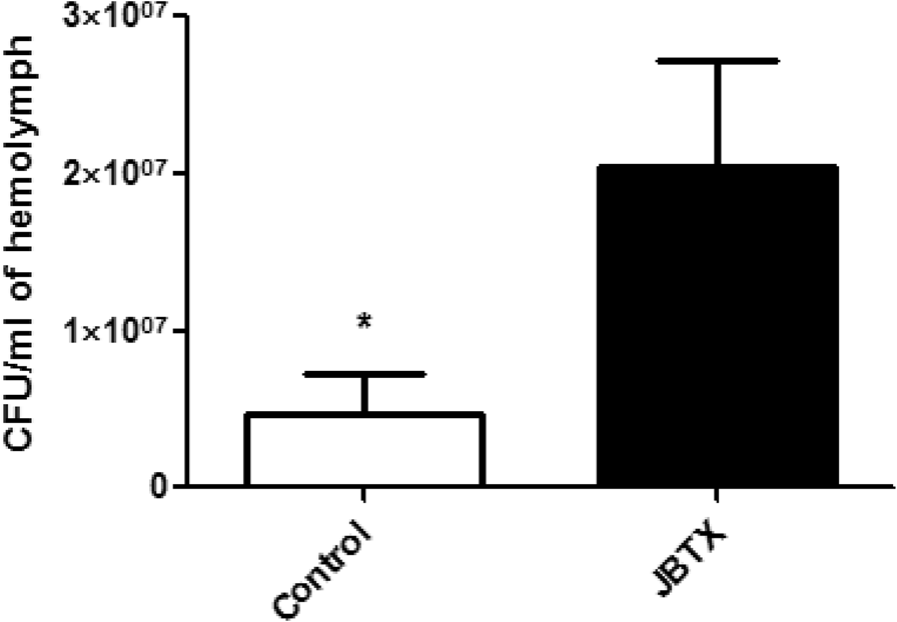
Jaburetox injections diminished the clearance of bacteria from *R. prolixus* hemolymph. Fifth-instar nymphs (7 days after ecdysis) were injected with PB (controls) or with Jaburetox in a dose of 2 μg/insect. Five hours later, both groups were injected with ~1.8 × 10^6^ CFU of *Staphylococcus aureus* ATCC 25923. Five hours afterwards, the number of CFU in each insect’s hemolymph was estimated by the drop-plate method. The values are expressed as the number of CFU per ml of hemolymph. Mean ± SEM (*n* = 11). *P < 0.05 *vs* control

Fifth-instar nymphs were injected with PB (controls) or with Jaburetox and 5 h afterwards both groups were inoculated with ~1.8 × 10^6^ CFU of the pathogenic bacteria *Staphylococcus aureus*. Five hours later, the hemolymph was collected and the bacterial CFU was determined. In the Jaburetox-treated insects the clearance of bacteria from the hemolymph was significantly reduced as compared to that of control insects injected with PB (Mann-Whitney U = 28.00, *P =* 0.035) (Fig. 10). Moreover, only the Jaburetox group exhibited mortality after 10 h, which reached ~30 %. The gram-positive bacteria *S. aureus* used in this experiment is known to be pathogenic for insects [68–70]. Azambuja et al. [71] and Feder et al. [37] reported that in *R. prolixus*, upon a bacterial inoculation*,* a coordinated immune response is induced involving the production of antimicrobial peptides, increase in PO activity and formation of nodules. The rate of successful clearance of bacteria from the hemolymph depends on the number of inoculated cells and the physiological state of the insect. In our model, it is likely that the in vivo activation of the immune system induced by Jaburetox rendered the insects more susceptible to bacteria proliferation because it consumes defense resources that cannot be later employed to withstand the infection. For instance, the hemocytes that are engaged in aggregation as consequence of the previous Jaburetox injection are not available for a subsequent bacterial challenge due to the irreversible nature of the binding involved in nodulation [72]. This could have an impact in the insect’s capacity to clear bacteria even though the number of free hemocytes is not significantly modified upon Jaburetox treatment (Figs. 2, 3, 4 and 5). Noteworthy, not all hemocytes show phagocytic and aggregating activity against bacteria [73]. Despite the fact that PO activity levels were higher in insects injected with Jaburetox at the moment of bacteria inoculation, this may not be enough for an effective bacterial clearance. It is also possible that the early activation of the PO activity causes a depletion of the enzyme in the hemocytes [74] affecting the bacterial elimination system as the time progresses. Moreover, additional aspects of the immune response could be altered by the Jaburetox treatment and we are currently exploring these possibilities.

## Conclusions

A combination of biochemical and cellular approaches led us to conclude that Jaburetox triggers a cellular immune response in *R. prolixus*, both in vivo and in vitro. Mediator(s) of the COX pathway participate in this process, although a contribution of the LOX products cannot be ruled out. Jaburetox also elicits a humoral immune response, since PO activity levels are significantly increased upon the injection of the toxic peptide. On the other hand, Jaburetox does not enhance the antibacterial response, as unchanged hemolymph levels of lysozyme, cecropin and turbidimetric assays of bacterial growth were seen; and also as Jaburetox-treated insects were more susceptible to bacterial infection, indicative of immunosuppression. Moreover, the toxic peptide causes a dose-dependent cytoskeleton damage, chromatin fragmentation and apoptosis induction in cultured hemocytes, without modifying their phagocytic activity.

The immune responses prompted by Jaburetox in *R. prolixus* such as hemocyte aggregation and PO increased activity, are also triggered by pathogens (bacteria and parasites for instance), raising the possibility that the toxic peptide or some processed form of it would be recognized by the defense system as a PAMP. Jaburetox targets several organs and tissues in insects, including the digestive and central nervous system, Malpighian tubules and salivary glands. Taken together with other results from our group, the present data contribute to a general picture of the entomotoxic mode of action of Jaburetox and lay foundations for new, in-depth studies. Some of the questions that we are currently trying to answer are: Does the activation of the proPO cascade indicate that Jaburetox is recognized as a PAMP? How is Jaburetox processed by cultured cells? These and other questions are being addressed and hopefully would lead us closer to a better comprehension of this toxin and of *R. prolixus’* physiology.

## Abbreviations

baic, baicalein; CFU, colony-forming units; COX, cyclooxygenases; DAPI, 4′,6-diamidino-2-phenylindole; dexa, dexamethasone; escu, esculetin; FBS, fetal bovine serum; FITC, Fluorescein Isothiocyanate; indo, indomethacin; JBU, jack bean urease; LB, Luria-Bertani; LOX, lipoxygenases; Na2EDTA, ethylenediaminetetraacetic acid, disodium salt; NO, nitric oxide; NOS, nitric oxide synthase; PAMP, pathogen associated molecular pattern; PB, sodium phosphate buffer; PLA2, phospholipase A2; PO, phenoloxidase; proPO, prophenoloxidase; RFU, relative fluorescence units; RT, room temperature; SEM, standard error of the mean; UAP, UDP-N-acetylglucosamine pyrophosphorylase

## Additional file

Additional file 1: Figure S1. Antibacterial cecropin-like activity in plates seeded with *Escherichia coli* ATCC 25922. Two μl of each sample were added in holes punched in the agar plate and the inhibition zone were recorded after and incubation of 24 h at room temperature. 1: hemolymph from PB-injected insects (negative control); 2: hemolymph from Jaburetox-injected insects; 3: cecropin A (0.25 μg/μl, positive control); 4: hemolymph from insects injected with ~1 × 10^6^ *E coli* live cells (positive control). (TIF 2229 kb)
