# Supplementary figures and images for: Humoral and cellular immune responses induced by the urease-derived peptide Jaburetox in the model organism Rhodnius prolixus
Source: Parasit Vectors. 2016 Jul 25;9:412. doi: 10.1186/s13071-016-1710-3 (PMC4960889; doi:10.1186/s13071-016-1710-3)

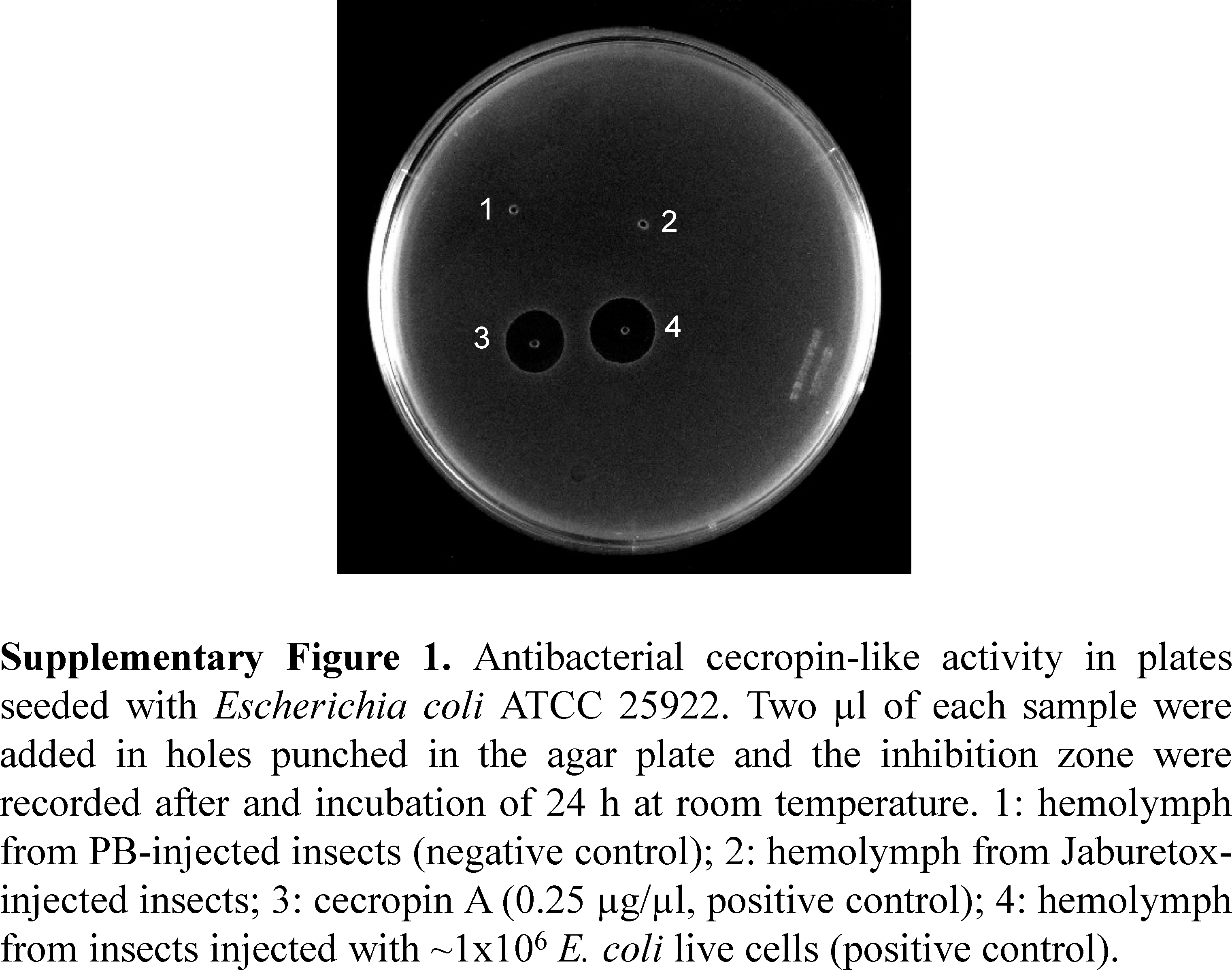

Supplement: Additional file 1: Figure S1. — Antibacterial cecropin-like activity in plates seeded with Escherichia coli ATCC 25922. Two μl of each sample were added in holes punched in the agar plate and the inhibition zone were recorded after and incubation of 24 h at room temperature. 1: hemolymph from PB-injected insects (negative control); 2: hemolymph from Jaburetox-injected insects; 3: cecropin A (0.25 μg/μl, positive control); 4: hemolymph from insects injected with ~1 × 10^6^ E coli live cells (positive control). (TIF 2229 kb) [file 13071_2016_1710_MOESM1_ESM.tif]
